# Neoadjuvant Bevacizumab Plus Docetaxel/Cisplatin/Capecitabine Chemotherapy in Locally Advanced Gastric Cancer Patients: A Pilot Study

**DOI:** 10.3389/fsurg.2022.842828

**Published:** 2022-05-11

**Authors:** Deguo Yu, Zhenfeng Wang, Tingbang He, Lijun Yang

**Affiliations:** ^1^Department of Emergency Surgery, The Second People's Hospital of Liaocheng, Linqing, China; ^2^Department of General Surgery, The Second People's Hospital of Liaocheng, Linqing, China; ^3^Department of General Surgery, The People's Hospital of XiaJin Affiliated to Shandong First Medical University, Xiajin, China; ^4^Department of Emergency, The Second People's Hospital of Liaocheng, Linqing, China

**Keywords:** Bevacizumab plus chemotherapy, neoadjuvant regimen, treatment response, survival data, adverse events

## Abstract

**Background:**

Bevacizumab (BEV) plus chemotherapy as a neoadjuvant regimen presents good efficacy in patients with locally advanced cancer. However, its role in patients with locally advanced gastric cancer (LAGC) is not clear. Thus, the study aimed to assess the efficacy and safety of neoadjuvant BEV plus chemotherapy in patients with LAGC.

**Methods:**

Twenty resectable patients with LAGC who received BEV plus docetaxel/cisplatin/capecitabine (DCC) chemotherapy for 3 cycles with 21 days as one cycle as neoadjuvant regimen were involved. Besides, their treatment response, survival profiles, and adverse events were assessed.

**Results:**

In total, two (10.0%), 9 (45.0%), 8 (40.0%), and 1 (5.0%) patients achieved complete remission, partial remission, stable disease, and progressive disease (PD) according to imaging evaluation, which resulted in 55.0% of objective response rate and 95.0% of disease control rate, respectively. Moreover, the number of patients with pathological response grades 1, 2, and 3 was 8 (40.0%), 8 (40.0%), and 3 (15.0%); while 1 (5.0%) patient did not receive surgery due to PD, thus the data of this patient was not assessable. Meanwhile, 18 (90.0%) patients achieved R0 resection. Regarding survival profile, the median disease-free survival or overall survival were both not reached. The 1-year, 2-, and 3-year disease-free survival rates were 88.8, 80.7, and 67.3%. Meanwhile, the 1-, 2-, and 3-year overall survival rates were 100.0%, 75.8%, and 75.8%, respectively. Additionally, the main adverse events were anemia (90.0%), alopecia (90.0%), leukopenia (70.0%), and anorexia (65.0%). Indeed, most adverse events were of grade 1 or 2 and were manageable.

**Conclusion:**

Neoadjuvant BEV plus DCC chemotherapy presents a favorable pathological response and survival profile with acceptable safety in patients with LAGC.

## Introduction

Gastric cancer is one of the most common malignancies worldwide, which affects ~990,000 people and causes around 738,000 deaths every year (1–3). In China, the incidence of gastric cancer is ~30.64 per 100,000 populations per year (4). In all gastric cancer, locally advanced gastric cancer (LAGC) is a special type since the prognosis of patients with LAGC is dramatically different between resectable and unresectable ones (5–7); therefore, creating surgery opportunities for the patients with unresectable LAGC by neoadjuvant chemotherapy is necessary (8–11). Meanwhile, it is also critical to optimize surgical conditions for the patients with resectable LAGC by neoadjuvant chemotherapy, which reduces postoperative recurrence, thus further improving survival in these patients (12–14). Among the regimens of neoadjuvant chemotherapy, docetaxel, cisplatin, and capecitabine (DCC), chemotherapy is tolerable and brings promising efficacy with a 5-year survival rate of 54% in R0 patients with resected LAGC (15).

Bevacizumab (BEV), an angiogenesis inhibitor, binds to vascular endothelial growth factor (VEGF)-A to prevent the interaction of VEGF-A with the VEGF receptor, then suppresses the VEGF signaling pathway, thereby inhibiting neovascularization (16, 17). Nowadays, BEV plus chemotherapy as a neoadjuvant regimen is used in patients with locally advanced cancers (14, 18, 19). For instance, neoadjuvant BEV plus oxaliplatin, leucovorin, and 5-fluorouracil realize a pathological downstaging rate of 65% in locally advanced rectal cancer patients (14); moreover, another research illustrates that BEV plus paclitaxel and carboplatin as a neoadjuvant regimen achieves 100% objective response rate (ORR) and an optimal pathological response of 38% in patients with locally advanced cervical cancer (18); besides, as for unresectable stage III lung adenocarcinoma patients, neoadjuvant BEV plus pemetrexed and carboplatin induces pathologic downstaging rate of 73.8% and 1-year event-free survival rate of 56.1% (19). Based on the above-mentioned information, we hypothesized that BEV plus chemotherapy as neoadjuvant therapy might be a promising regimen in LAGC, while it is rarely applied in LAGC.

The current pilot study aimed to investigate the efficacy and safety of neoadjuvant BEV plus DCC in patients with resectable LAGC.

## Methods

### Patients

This study serially recruited twenty patients with LAGC who were about to receive BEV plus DCC as a neoadjuvant regimen from July 2017 to December 2019. The patients were recruited in the study if they met the following criteria: (i) diagnosed as gastric adenocarcinoma pathologically and histologically; (ii) over 18 years old; (iii) clinical tumor-node-metastasis (cTNM) stage III (cT3 to cT4a, cN+, and cM0) according to the eighth edition of TNM classification (20); (iv) Eastern Cooperative Oncology Group (ECOG) score 0 to 1; (v) with resectable tumor; (vi) about to receive BEV plus DCC as a neoadjuvant regimen. The patients were excluded from the study if they had any of the following conditions: (i) had other carcinoma or malignancy; (ii) allergic to the drugs used in the study; (iii) unwilling to be followed up regularly; (iv) during pregnancy or breastfeeding. The study was approved by the Institutional Review Board. All patients provided written informed consents.

### Treatment

The patients underwent BEV plus DCC as a neoadjuvant regimen for 3 cycles with 21 days as one cycle. At 4 weeks after the end of the last neoadjuvant therapy cycle, the tumor resectability was evaluated again based on the CT examinations in terms of the Japanese classification of gastric carcinoma (21), then the surgical resection was performed if the patient's tumor was deemed resectable. At 4–8 weeks after surgery, the patients continued to receive DCC adjuvant therapy for 3 cycles depending on the patient's recovery. The recommended regimen of neoadjuvant therapy was as followed: BEV was administered intravenously at the dose of 7.5 mg/kg on day 1; docetaxel was administered intravenously at the dose of 60 mg/m^2^ on day 1; cisplatin was administered intravenously at the dose of 60 mg/m^2^ on day 1; capecitabine was administered orally at the dose of 937.5 mg/m^2^ twice daily from day 1 to day 14 (22). The specific dose of the above regimen was allowed to be adjusted depending on the patients' response and tolerance.

### Outcome Assessment

At 4 weeks after the end of the last neoadjuvant therapy cycle, clinical response was evaluated based on CT examinations according to the Response Evaluation Criteria in Solid Tumors (RECIST) (23), including complete remission (CR), partial remission (PR), stable disease (SD), and progressive disease (PD), based on which, the ORR and the disease control rate (DCR) were calculated. During surgery, the pathological response was assessed based on intraoperative pathological examinations in accordance with the Japanese classification of gastric carcinoma (21), which was classified into four grades: (i) grade 0, there was no evidence of effect; (ii) grade 1, there were viable tumor cells (the cells judged to be capable of proliferating) in more than 1/3 of the tumor areas; (iii) grade 2, there were viable tumor cells in <1/3 of the tumor areas; (iv) grade 3, there were no viable tumor cells in the tumor areas. After surgery, the R0 resection rate was evaluated based on the resection margin of formalin-fixed paraffin-embedded (FFPE) tumor specimens, and R0 resection was defined as the resection without remaining macroscopic or microscopic residual lesion. In addition, adverse events were recorded to assess the treatment safety and were graded in terms of the National Cancer Institute Common Terminology Criteria for Adverse Events (CTCAE, version 4.0).

### Follow-Up

All patients were followed up regularly until January 2021, and the median follow-up period was 20.1 months with the range of 7.5–36.1 months. On the basis of the follow-up data, disease-free survival (DFS) and overall survival (OS) were calculated. DFS was defined as the duration from surgery to the disease relapse or the patient's death; OS was defined as the duration from surgery to the patient's death (24).

### Statistical Analysis

Count data were expressed as percentages, and measurement data were presented as mean ± SD. DFS and OS were constructed with the Kaplan-Meier curves. SPSS 26.0 (IBM Corp., Armonk, New York, USA) and GraphPad Prism 7.02 (GraphPad Software Inc., San Diego, California, USA) were applied for statistical analysis and figure plotting, respectively.

## Results

### Patients' Characteristics

A total of 20 patients with LAGC were enrolled in the study, who presented a mean age of 58.1 ± 9.8 years with 8 (40.0%) females and 12 (60.0%) males. In terms of tumor differentiation, 1 (5.0%) patient was of good differentiation, 6 (30.0%) patients were of moderate differentiation, and 13 (65.0%) patients were of poor differentiation. About the cT stage, the number of patients with cT3 stage and cT4a stage was 5 (25.0%) and 15 (75.0%) respectively. As for the cN stage, there were 7 (35.0%) patients with cN1 stage, 7 (35.0%) patients with cN2 stage, and 6 (30.0%) patients with cN3 stage. The detailed clinical features were shown in Table 1.

**Table 1:** Clinical characteristics.

**Items**	**LAGC patients (*N* = 20)**
Age (years), mean±SD	58.1 ± 9.8
Gender, No. (%)	
Female	8 (40.0)
Male	12 (60.0)
Current smoke, No. (%)	6 (30.0)
Current drink, No. (%)	7 (35.0)
Hypertension, No. (%)	5 (25.0)
Hyperlipidemia, No. (%)	3 (15.0)
Diabetes, No. (%)	2 (10.0)
H.pylori infection, No. (%)	
Negative	13 (65.0)
Positive	7 (35.0)
Tumor location, No. (%)	
Cardia	8 (40.0)
Gastric body	9 (45.0)
Gastric antrum	3 (15.0)
Differentiation, No. (%)	
Well	1 (5.0)
Moderate	6 (30.0)
Poor	13 (65.0)
cT stage, No. (%)	
cT3	5 (25.0)
cT4a	15 (75.0)
cN stage, No. (%)	
cN1	7 (35.0)
cN2	7 (35.0)
cN3	6 (30.0)

*LAGC, locally advanced gastric cancer; SD, standard deviation; H.pylori, helicobacter pylori; cT, clinical tumor; cN, clinical node*.

### Treatment Response

**Figure 1:**
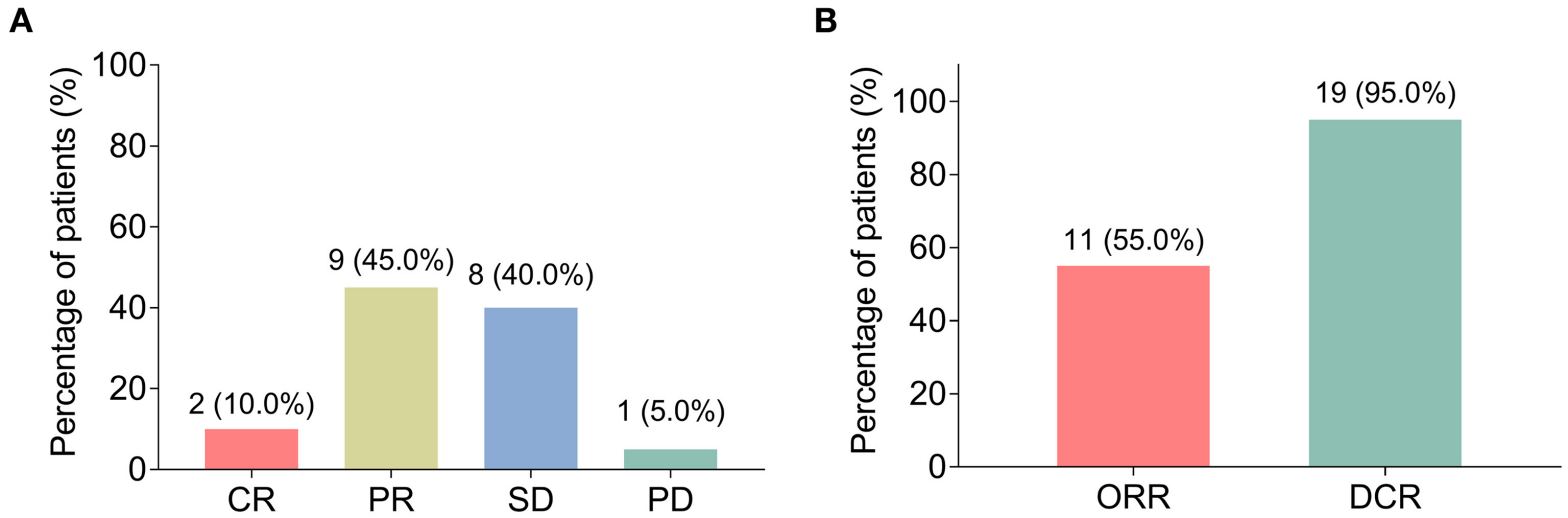
Clinical response. The percentage of patients with locally advanced gastric cancer (LAGC) with complete remission (CR), partial remission (PR), stable disease (SD), and progressive disease (PD) **(A)**; the percentage of LAGC patients with objective response rate (ORR) and disease control rate (DCR) **(B)**.

All patients completed 3 cycles of BEV plus chemotherapy as neoadjuvant therapy, and no patient violated the protocol due to side effects. After neoadjuvant therapy, 2 (10.0%) patients achieved CR, 9 (45.0%) patients achieved PR, 8 (40.0%) patients had SD, and 1 (5.0%) patient had PD (Figure 1A). Therefore, the ORR (CR+PR) was 55.0% and the DCR (CR+PR+SD) was 95.0% (Figure 1B).

**Figure 2:**
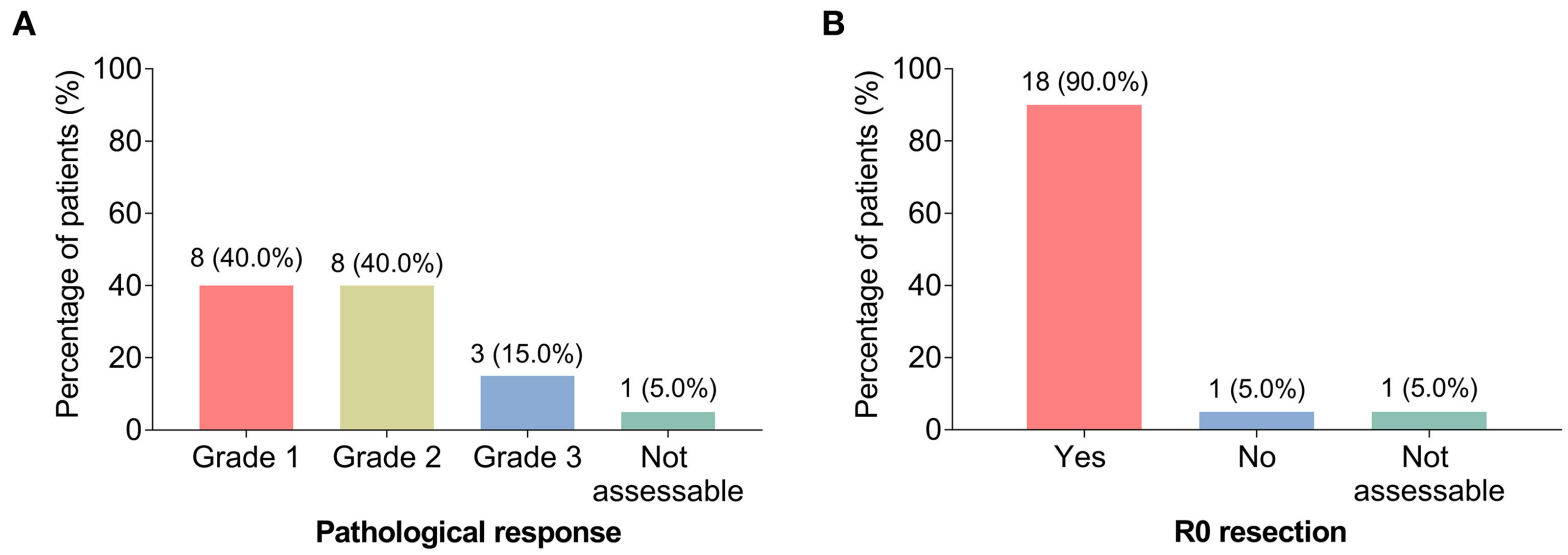
Pathological response and R0 resection. The percentage of patients with LAGC with grade 1, grade 2, and grade 3 pathological response **(A)**; the percentage of patients with LAGC with R0 resection and without R0 resection **(B)**.

During surgery, the pathological response was evaluated. The number of patients with pathological response grades 1, 2, and 3 were 8 (40.0%), 8 (40.0%), and 3 (15.0%); while 1 (5.0%) patient did not receive surgery due to PD, thus the data of this patient was not assessable (Figure 2A).

After surgery, R0 resection was also assessed, which showed that 18 (90.0%) patients achieved the criteria, while only 1 (5.0%) patient did not achieve it; the data of 1 (5.0%) patient was not assessable for the same reason mentioned above (Figure 2B).

### DFS and OS

**Figure 3:**
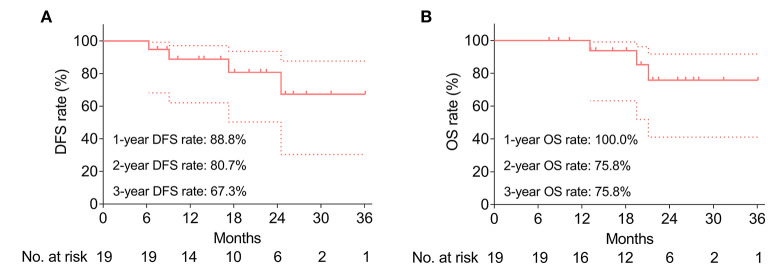
Survival profile. Accumulating DFS rate **(A)** and accumulating OS rate **(B)**. Dotted line represents the 95% CI of DFS or OS.

During a median follow-up of 20.1 months (range: 7.5–36.1 months), the median DFS and OS were both not reached. The 1-, 2-, and 3-year DFS rates were 88.8%, 80.7%, and 67.3%, respectively (Figure 3A). Meanwhile, the 1-, 2-, and 3-year OS were 100.0%, 75.8%, and 75.8%, respectively (Figure 3B). Besides, the key characteristics and treatment outcomes of each patient were presented in Table 2.

**Table 2:** Key characteristics and treatment outcomes of each patient.

**No**.	**Age** **(years)**	**Gender**	**Tumor location**	**Differentiation**	**cT stage**	**cN stage**	**Clinical** **response**	**Resection**	**R0 resection**	**Pathological** **response**	**Relapse**	**DFS** **(months)**	**Death**	**OS** **(months)**
1	63	Female	Cardia	Moderate	cT4a	cN3	SD	Yes	Yes	Grade 1	No	21.7	No	21.7
2	67	Female	Gastric antrum	Poor	cT3	cN1	CR	Yes	Yes	Grade 3	No	36.1	No	36.1
3	46	Male	Cardia	Moderate	cT4a	cN2	PR	Yes	Yes	Grade 2	No	25.1	No	25.1
4	52	Male	Gastric body	Poor	cT3	cN1	SD	Yes	Yes	Grade 2	No	10.3	No	10.3
5	57	Male	Gastric body	Poor	cT4a	cN2	PR	Yes	Yes	Grade 2	No	8.8	No	8.8
6	56	Female	Cardia	Moderate	cT3	cN1	CR	Yes	Yes	Grade 3	No	31.4	No	31.4
7	46	Female	Gastric body	Well	cT4a	cN3	PR	Yes	Yes	Grade 1	No	7.5	No	7.5
8	63	Male	Gastric body	Poor	cT4a	cN1	PR	Yes	Yes	Grade 2	No	16.2	No	16.2
9	72	Female	Cardia	Poor	cT4a	cN1	SD	Yes	Yes	Grade 1	Yes	6.3	Yes	13.1
10	45	Male	Gastric body	Poor	cT4a	cN3	SD	Yes	Yes	Grade 1	No	18.1	No	18.1
11	69	Male	Cardia	Poor	cT4a	cN1	SD	Yes	Yes	Grade 1	Yes	24.5	No	27.3
12	61	Male	Gastric body	Poor	cT3	cN1	PR	Yes	Yes	Grade 2	No	28.0	No	28.0
13	64	Male	Gastric antrum	Poor	cT4a	cN3	PD	No	-	-	-	-	-	-
14	57	Male	Cardia	Moderate	cT4a	cN2	PR	Yes	Yes	Grade 1	No	22.5	No	22.5
15	68	Male	Cardia	Poor	cT4a	cN3	SD	Yes	No	Grade 1	Yes	9.1	Yes	19.5
16	43	Male	Gastric body	Moderate	cT4a	cN3	PR	Yes	Yes	Grade 2	No	26.2	No	26.2
17	68	Female	Gastric antrum	Moderate	cT4a	cN2	PR	Yes	Yes	Grade 2	No	20.1	No	20.1
18	60	Male	Gastric body	Poor	cT3	cN2	PR	Yes	Yes	Grade 3	No	13.9	No	13.9
19	65	Female	Gastric body	Poor	cT4a	cN2	SD	Yes	Yes	Grade 2	Yes	17.3	Yes	21.1
20	39	Female	Cardia	Poor	cT4a	cN2	SD	Yes	Yes	Grade 1	No	13.2	No	13.2

*cT, clinical tumor; cN, clinical node; DFS, disease-free survival; OS, overall survival; SD, stable disease; CR, complete remission; PR, partial remission; PD, progressive disease*.

### Adverse Events

The most common hematological adverse events were anemia (90.0%), leukopenia (70.0%), neutropenia (60.0%), and thrombocytopenia (35.0%); meanwhile, the most frequent non-hematological adverse events were alopecia (90.0%), anorexia (65.0%), and fatigue (45.0%) (Table 3). Besides, most adverse events were of grade 1 or grade 2. Additionally, grade 3 adverse events were anemia (15.0%), leukopenia (20.0%), neutropenia (25.0%), thrombocytopenia (5.0%), anorexia (5.0%), diarrhea (5.0%), and elevated transaminase (5.0%).

**Table 3:** Adverse events.

**Items**	**Total**	**Grade 1**	**Grade 2**	**Grade 3**	**Grade 4**
**Hematological adverse events**					
Anemia, No. (%)	18 (90.0)	10 (50.0)	5 (25.0)	3 (15.0)	0 (0.0)
Leukopenia, No. (%)	14 (70.0)	7 (35.0)	3 (15.0)	4 (20.0)	0 (0.0)
Neutropenia, No. (%)	12 (60.0)	5 (25.0)	2 (10.0)	5 (25.0)	0 (0.0)
Thrombocytopenia, No. (%)	7 (35.0)	4 (20.0)	2 (10.0)	1 (5.0)	0 (0.0)
**Non-hematological adverse events**					
Alopecia, No. (%)	18 (90.0)	14 (70.0)	4 (20.0)	0 (0.0)	0 (0.0)
Anorexia, No. (%)	13 (65.0)	8 (40.0)	4 (20.0)	1 (5.0)	0 (0.0)
Fatigue, No. (%)	9 (45.0)	6 (30.0)	3 (15.0)	0 (0.0)	0 (0.0)
Nausea and vomiting, No. (%)	8 (40.0)	5 (25.0)	3 (15.0)	0 (0.0)	0 (0.0)
Diarrhea, No. (%)	6 (30.0)	4 (20.0)	1 (5.0)	1 (5.0)	0 (0.0)
Hand-foot syndrome, No. (%)	5 (25.0)	2 (10.0)	3 (15.0)	0 (0.0)	0 (0.0)
Hypertension, No. (%)	4 (20.0)	3 (15.0)	1 (5.0)	0 (0.0)	0 (0.0)
Elevated transaminase, No. (%)	4 (20.0)	2 (10.0)	1 (5.0)	1 (5.0)	0 (0.0)
Constipation, No. (%)	3 (15.0)	2 (10.0)	1 (5.0)	0 (0.0)	0 (0.0)
Pruritus, No. (%)	3 (15.0)	2 (10.0)	1 (5.0)	0 (0.0)	0 (0.0)
Proteinuria, No. (%)	3 (15.0)	1 (5.0)	2 (10.0)	0 (0.0)	0 (0.0)

## Discussion

Neoadjuvant chemotherapy has been extensively applied in patients with LAGC (25, 26). For instance, neoadjuvant DCC shows an R0 resection rate of 86.7% and R1 resection rate of 4.5% in patients with resectable gastric cancer (25); in patients with LAGC, neoadjuvant docetaxel, cisplatin, and S-1 realizes a partial response rate of 57% and an SD rate of 43%; meanwhile, it also facilitates a pathological response grade 1a in 17% patients, grade 1b in 30% patients, grade 2 in 37% patients and grade 3 in 17% patients (26). Recently, a trial reports that BEV plus DCC as neoadjuvant regimen presents pleasing treatment response in previous patients with unresectable LAGC or paraaortic lymph node metastatic gastric cancer, and the data shows that the ORR is 64.3%; meanwhile, the pathological complete regression rate is 12.9% (22), which indicates that neoadjuvant BEV plus DCC may improve treatment response in patients with gastric cancer. Thus, our study further explored the role of neoadjuvant BEV plus DCC in patients with LAGC and discovered that BEV plus DCC as neoadjuvant therapy presented a good clinical response, with an ORR of 55.0% and a pathological response grade 2 in 40.0% of patients and grade 3 in 15.0% patients, which was numerically better than DCC chemotherapy alone as neoadjuvant therapy (25). The explanations might be that (1) BEV could inhibit the neovascularization and induce the regression of tumor blood vessels (16), meanwhile, chemotherapy exhibited the ability of anti-cancer cytotoxicity (27), therefore, BEV plus DCC might present a better anti-tumor effect; (2) BEV might enhance the chemosensitivity of gastric cancer cells *via* suppressing the VEGF- phosphatidylinositol 3 kinase/protein kinase B-survivin signaling cascade (28); thus, it combined with DCC could induce favorable outcomes.

Neoadjuvant chemotherapy has been reported to improve survival in patients with LAGC. For example, neoadjuvant DCC chemotherapy displays acceptable survival with the median progression-free survival (PFS) and OS of 12.1 months (range 9.5–14.6 months) and 22.9 months (range 14.3–31.5 months) in patients with unresectable LAGC (15); docetaxel plus S-1 as neoadjuvant regimen shows a high 3-year PFS rate compared with surgery alone (80.0 vs. 58.7%) in patients with LAGC (29); patients with LAGC receiving neoadjuvant epirubicin, oxaliplatin, and capecitabine chemotherapy present PFS rate of 40% and OS rate of 64.4% over a 3-year follow-up (30). In our study, BEV plus DCC as a neoadjuvant regimen for patients with LAGC displayed that the 1-, 2-, and 3-year DFS rates were 88.8, 80.7, and 67.3%, and the 1-, 2-, and 3-year OS were 100.0, 75.8, and 75.8%, which was numerically better than previous study using DCC alone as the neoadjuvant regimen (15). The results might be because BEV plus DCC presented more effective on tumor downstaging and pathological response than DCC chemotherapy alone (mentioned above), which directly reduced the recurrence risk to improve their prognosis.

The safety of BEV and DCC in patients with cancer has been reported. The main toxicities related to BEV are hypertension, proteinuria, and hemorrhage which can limit therapy and lead to other complications (31, 32). The most common adverse events after DCC treatment are neutropenia, anorexia, and febrile neutropenia (33). In the present study, the common hematological adverse events were anemia, leukopenia, and neutropenia, and the non-hematological adverse events were alopecia, anorexia, and fatigue. The incidences of adverse events were generally similar compared with a previous study (33). Besides, the adverse events were manageable, and most of them were of grades 1 and 2, which revealed that BEV plus DCC chemotherapy as neoadjuvant therapy was a tolerable option for patients with LAGC. However, further studies should be conducted to further verify the safety of BEV plus DCC as neoadjuvant therapy in patients with LAGC.

Some limitations still existed in our study: (1) we did not enroll patients with LAGC receiving neoadjuvant DCC chemotherapy as control, therefore, further randomized controlled trial could be performed; (2) the sample size of this study was only 20 patients because the usage of BEV as neoadjuvant therapy in patients with LAGC remained in exploring stage, thus, a subsequent study with larger sample size was needed to verify our conclusion; (3) the follow-up time was relatively short, which might affect the DFS and OS evaluation in statistics; (4) the effect of other anti-angiogenesis agents (such as apatinib) combining with chemotherapy as a neoadjuvant regimen in LAGC could be investigated further.

In conclusion, neoadjuvant BEV plus DCC presents a favorable pathological response and survival profile with acceptable safety in patients with LAGC. The results of this study highlight that BEV plus DCC is a potentially neoadjuvant regimen that may achieve survival benefits in patients with LAGC.

## Data Availability Statement

The original contributions presented in the study are included in the article/supplementary material, further inquiries can be directed to the corresponding author.

## Ethics Statement

The studies involving human participants were reviewed and approved by Institutional Review Board of the People's Hospital of XiaJin Affiliated to Shandong First Medical University. The patients/participants provided their written informed consent to participate in this study.

## Author Contributions

DY and TH contributed to the study design and manuscript writing. DY, ZW, TH, and LY conducted literature research and clinical practice. DY, ZW, and TH contributed to the data acquisition and analysis. DY and TH reviewed the manuscript and made revisions. All authors read and approved the final manuscript. The figures were made by the authors' own work. All authors contributed to the article and approved the submitted version.

## Conflict of Interest

The authors declare that the research was conducted in the absence of any commercial or financial relationships that could be construed as a potential conflict of interest.

## Publisher's Note

All claims expressed in this article are solely those of the authors and do not necessarily represent those of their affiliated organizations, or those of the publisher, the editors and the reviewers. Any product that may be evaluated in this article, or claim that may be made by its manufacturer, is not guaranteed or endorsed by the publisher.
